# Bilateral Posterior Vitreous Detachment in a Young Patient with Systemic Lupus Erythematosus

**DOI:** 10.7759/cureus.2692

**Published:** 2018-05-26

**Authors:** Zain Abid, Sidra Khan, Sana Kaleem, Ali M Tahir, Ujala Zubair

**Affiliations:** 1 Department of Oncology, Jinnah Postgraduate Medical Center, Karachi, PAK; 2 Internal Medicine, Jinnah Postgraduate Medical Center, Karachi, PAK; 3 Internal Medicine, Jinnah Postgraduate Medical Center, karachi, PAK; 4 Medicine, Dow University of Health Sciences (DUHS), Karachi, Pakistan

**Keywords:** vitreous detachment, systemic lupus erythematous

## Abstract

Vitreous detachment is a rarely reported manifestation of systemic lupus erythematosus (SLE). We present here the case of a 20-year-old woman with a history of SLE who presented with a decrease in vision in both eyes, massive hepatosplenomegaly, and multiple joint pain. B-scan ultrasonography revealed bilateral vitreous detachment. Ocular involvement in SLE is associated with high disease activity and morbidity. Therefore, ophthalmological screening for ocular involvement is important for SLE patients at any age.

## Introduction

Systemic lupus erythematosus (SLE) is a chronic autoimmune multisystem disease with ocular involvement in one-third of patients [1]. The ocular manifestations of SLE include mucocutaneous involvement of the eyelids, secondary Sjogren’s syndrome, optic neuropathy, retinal detachment, and vitreous hemorrhage [2]. Retinal pathology is present in 7%-27% of patients with active SLE. Ocular manifestations of SLE are due to the deposition of immune complexes in the basement membrane of small blood vessels [2], and they are associated with the presence of antiphospholipid antibodies, other neurologic manifestations, and increased mortality [3].

## Case presentation

A 20-year-old woman presented with complaints of fever, abdominal pain, and blurred vision in both eyes. She was diagnosed with SLE three years before presentation and was managing her condition with oral prednisone and hydroxychloroquine. A general physical examination was performed followed by radiological, biochemical, and ophthalmological examinations. Informed consent was taken before sharing her case with this academic journal.

The eyelids and conjunctiva of her right eye were unremarkable. We noted endothelial dusting on the posterior surface of her cornea. The refractive error and visual acuity of the right eye were 6/18 + 1.0 DS. The right pupil was not dilated due to the presence of posterior synechiae; fundus details were hazy and not completely visible due to dense vitreous haze. We noted no active vitreitis, but observed complete posterior vitreous detachment. The intraocular pressure of the right and left eyes was 12 mmHg. Both eyes had keratic precipitate. A cataract extraction was performed in the right eye, and a posterior chamber intraocular lens was placed.

Refractive error and visual acuity of her left eye were 6/9 + 0.75 DS. We noted ectropion uvea and endothelial dusting. The left pupil was mid-dilated, and the macula and vessels were unremarkable. However, a hypopigmented lesion on the retina inferior to the inferior arcade was present along with a hyperpigmented choroidal lesion. In the left eye, we also noted cells in the anterior chamber and anterior vitreous.

The B-scan ultrasonography revealed bilateral vitreous detachment. Thyroid profile, detailed urine report, and echocardiography results were normal. Her erythrocyte sedimentation rate (150 mm/h) and C-reactive protein levels (33 mg/dL) were elevated. Her serum C3 and C4 levels were within the reference ranges. Anticardiolipin antibodies and lupus anticoagulant levels were unremarkable, and tests for viral markers for hepatitis B and C were negative. An abdominal ultrasonography revealed that her liver measured 19.4 cm and had a subtle echotexture; the hepatic veins were not dilated. Her spleen measured 17.5 cm with uniform echotexture, and we noted bilateral early medullary nephrocalcinosis with Grade I parenchymal changes. A color Doppler showed dilatation of the portal vein (1.3 mm) and the splenic vein (1 cm).

**Figure 1:**
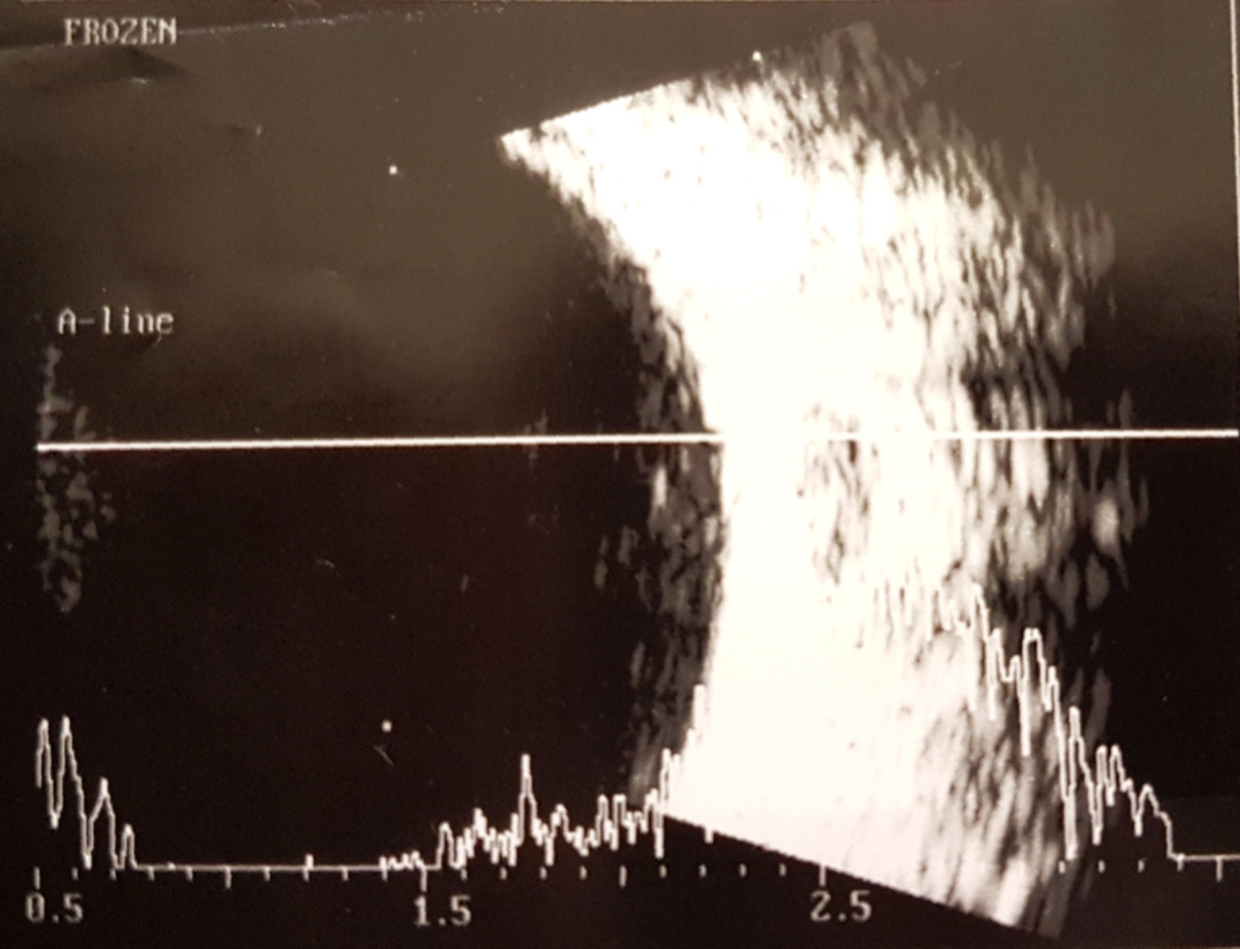
Optical coherence tomography scan.

**Figure 2:**
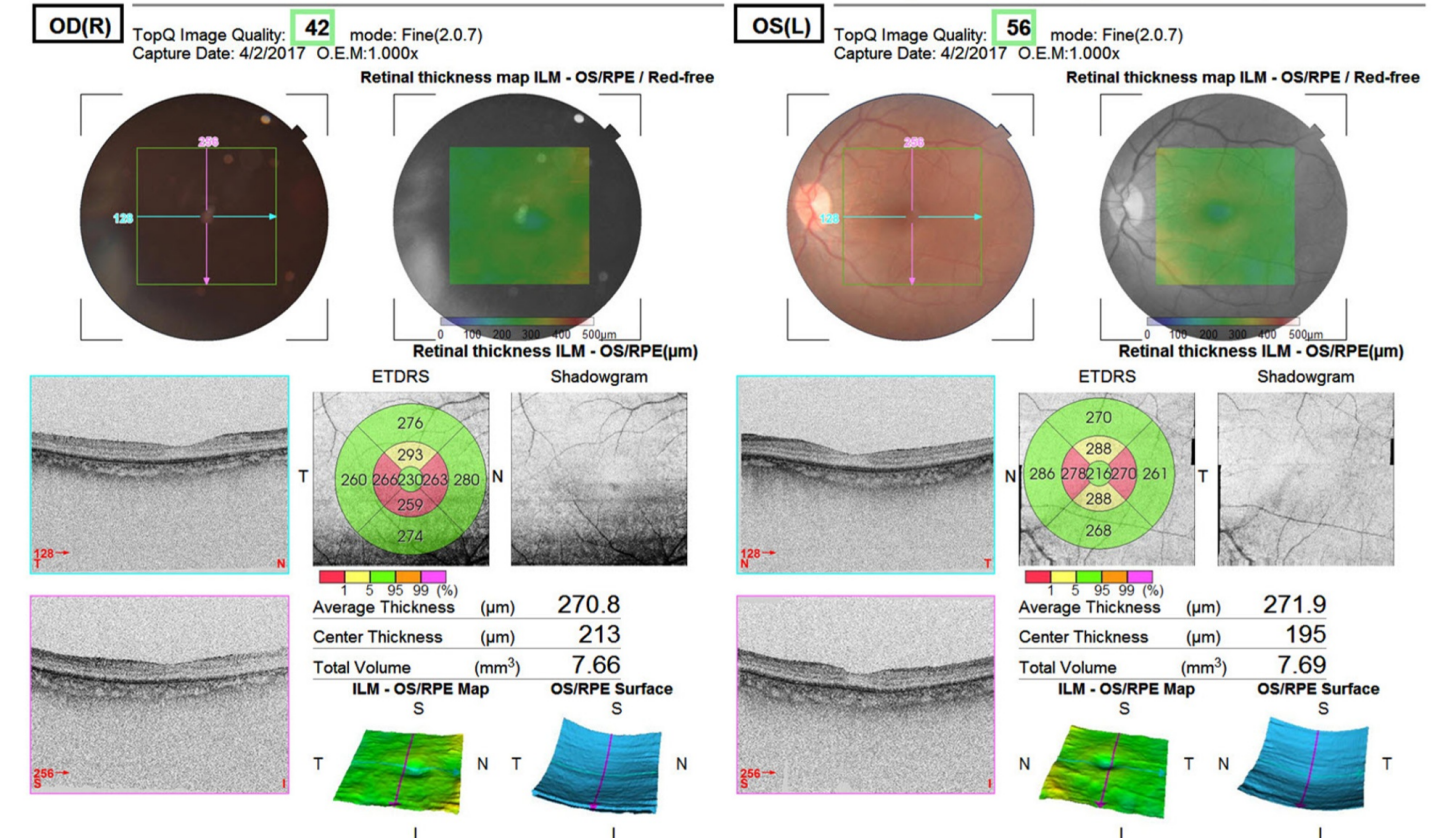
Ultrasonography B-scan.

Figure 1 shows the patient’s optical coherence tomography scan and Figure 2 presents the patient’s B-scan. In the right eye, the average retinal thickness is 270.8 µm, the central thickness is 213 µm, and the total volume is 7.66 mm^3^. In the left eye, the average retinal thickness is 271.9 µm, the central thickness is 195 µm, and the total volume is 7.69 mm^3^ (Figure 2).

## Discussion

Systemic lupus erythematosus is a chronic autoimmune inflammatory disorder with multisystem involvement. The diagnostic criteria for SLE do not include the presence of ocular manifestations. However, ocular manifestations of SLE reveal overall disease activity. In some scenarios, ocular manifestations of SLE indicate that the systemic manifestations are not being controlled adequately [4].

Hydroxychloroquine, used in the management of SLE, has the potential to cause retinal toxicity. Patients at risk for retinal toxicity include those with liver or kidney dysfunction, those receiving >6.5 mg/kg per day, those using hydroxychloroquine for more than five years, and elderly patients with or without pre-existing retinal disease [1].

Ocular manifestations occur in one-third of patients with SLE. However, vitreous detachment is a rare manifestation. No cases of bilateral vitreous detachment associated with SLE have been reported in the past 10 years. Mild to moderate retinal pathology is common, but posterior segment pathology such as vitreous detachment is relatively rare. In such cases, methylprednisone pulsed therapy and cyclophosphamide are useful treatments [5].

Other sight-threatening ocular manifestations of SLE include vitreous hemorrhage, optic atrophy, and proliferative retinopathy which can lead to glaucoma. These manifestations should be diagnosed and managed at an early stage to avoid future complications and impaired vision [6].

The management of retinal disease associated with SLE includes vigorous management of systemic disease with corticosteroids and immunosuppressants. In some conditions, ocular manifestations may require periocular steroid therapy, retinal surgery, or photocoagulation involving laser therapy [7].

## Conclusions

The presence of ocular involvement in a patient with SLE is associated with high disease activity and morbidity. Early screening for eye involvement by an ophthalmologist is important for SLE patients of any age.
